# Norwegian Physicians’ Knowledge of the Prices of Pharmaceuticals: A Survey

**DOI:** 10.1371/journal.pone.0075218

**Published:** 2013-09-11

**Authors:** Ida Iren Eriksen, Hans Olav Melberg, Berit Bringedal

**Affiliations:** 1 University of Oslo, Department of Health Management and Health Economics, Oslo, Norway; 2 LEFO, the Institute for Studies of the Medical Profession, Oslo, Norway; University of British Columbia, Canada

## Abstract

The objectives of this study are to measure physicians’ knowledge of the prices of pharmaceuticals, and investigate whether there are differences in knowledge of prices between groups of physicians. This article reports on a survey study of physicians’ knowledge of the prices of pharmaceuticals conducted on a representative sample of Norwegian physicians in the autumn of 2010. The importance of physicians’ knowledge of costs derives from their influence on total spending and allocation of limited health-care resources. Physicians are important drivers in the effort to contain costs in health care, but only if they have the knowledge needed to choose the most cost-effective treatment options. A survey was sent to 1 543 Norwegian physicians, asking them for price estimates and their opinions on the importance of considering the cost of treatment to society as a decision factor when treating their patients. This article deals with a subsection in which the physicians were asked to estimate the price of five pharmaceuticals: simvastatin, alendronate (Fosamax), infliximab (Remicade), natalizumab (Tysabri) and escitalopram (Cipralex). The response rate was 65%. For all the five pharmaceuticals, more than 50% and as many as 83% gave responses that differed more than 50% from the actual drug price. The price of more expensive pharmaceuticals was underestimated, while the opposite was the case for less expensive medicines. The data show that physicians in general have poor knowledge of the prices of the pharmaceuticals they offer their patients. However, the physicians who frequently deal with a drug have better knowledge of its price than those who do not handle a medication as often. The data also suggest that those physicians who agree that cost of care to society is an important decision factor have better knowledge of drug prices.

## Introduction

In this article we report on a study of physicians’ knowledge of and attitudes to the cost of treatment. The results, which show that physicians have poor knowledge of prices, can be seen as a demonstration of a specific market failure. In Norway, neither the patient nor the physician has to pay for treatment. The cost is borne by society and thus is likely to be ignored [1].

The importance of physicians’ knowledge of costs derives from their influence on the allocation of limited health-care resources. Physicians play a key role in making decisions about expensive tests and treatments. Health-care legislation and much of public policy is based on the assumption that physicians are well informed about the cost of care. For instance, the American College of Physicians’ sixth edition of their ethics manual [2] states that: “Physicians, patient advocates, insurers, and payers […] should base allocations on medical need, efficacy, cost-effectiveness, and proper distribution of benefits and burdens in society.” In other countries, such as Norway, the advice has been made a legal requirement. The Health Personnel Act states that “health personnel shall ensure that the health care does not mean unnecessary loss of time or unnecessary expenses…” [3]. Cost containment is a major issue in health-care management [4]. The incentives to check prices and search for cheaper products in a traditional market are based on the expectation of monetary gain. However, as Phelps put it: “If medical care is insured (nearly) fully, then the consumer’s personal benefit from finding a lower price is small” [1]. Social insurance blurs the incentives to care about the cost of treatment, but few questions have been asked as to how the street-level bureaucrats of the health-care sector relate to costs. The size of the health-care budget is mainly driven by physicians’ treatment decisions; efficiency is hence a large part of the cost function in health care.

For a drug to be allowed on the Norwegian market a marketing authorisation from The Norwegian Medicines Agency (NMA) must be approved. In the process of approval the Agency decides whether the consumer needs a prescription to be allowed to purchase the drug, and whether the drug will be reimbursed by the state or not, and under what medical or other conditions. For a drug to be reimbursed to a patient, the medical condition must be chronic, that is, the need for treatment must endure for more than three months. All residents of Norway are covered by the national insurance scheme, which is tax-financed. They have to pay all costs relating to the treatment of chronic conditions up to EUR275 (rate NOK to EUR0,135 date 31.01.2013, www.xe.com). After the costs reach this ceiling, the state reimburses close to everything. Maximum prices are set by the NMA. Pharmacies are allowed to charge lower prices, but variations across the country are small. Maximum prices are calculated using an international referencing system, based on the average of the three lowest prices in nine reference countries (Australia, Belgium, Denmark, Finland, Germany, Ireland, the Netherlands, Sweden and the United Kingdom) [5]. Maximum prices per package are stated in the catalogue of drug information for pharmaceuticals available free of cost online and on paper to all Norwegian physicians. The drugs simvastatin, alendronate and escitalopram are all drugs reimbursed by the state according to this model and are prescribed and administered by GPs, while infliximab and natalizumab are used at the expense of the hospital treating the patient, and are prescribed and administered by specialists. All Norwegian hospitals are part of a procurement cooperation where reduced prices are obtained on pharmaceuticals used in hospital through tendering processes. These procurement agreements reduce the price of drugs consumed in hospitals by an average of about 25% [6].

The first subject we investigate here is whether physicians know the prices of treatments. In times of rapid developments and changes in the tools available for diagnosing and treating medical conditions, it becomes increasingly difficult to keep track of all options. Based on earlier literature, there is reason to expect that physicians in general have poor knowledge of the cost of treatments they offer their patients [7–18].

Given the general level of knowledge of prices, we examine whether groups of physicians differ from each other. Other researchers have also been interested in the relationship between knowledge of the cost of treatments and other variables [17,19]. Ernst et al. criticised earlier studies on the subject of physicians’ knowledge of costs as having limited generalisability, because the drugs included in questionnaires were not necessarily routinely prescribed by the responding physicians [16]. By comparing the frequency with which a physician prescribes a treatment with his knowledge of the price of that treatment, we will obtain a better description of physicians’ cost knowledge. From this we can more easily determine whether changes or interventions should be implemented. Thus, the first relationship we test is whether those who frequently prescribe a drug have better knowledge of the price of this medication than others. Further, we investigate whether those who reveal cost-conscious attitudes also have better knowledge of prices. Previous research suggests that physicians who value cost as a decision factor also choose more cost-effective alternatives [20]. Finally, we explore whether gender, age and other demographic variables affect price knowledge. These issues will be investigated using a subsection of a questionnaire in which physicians were asked to estimate the price of five pharmaceuticals: simvastatin, alendronate (Fosamax), infliximab (Remicade), natalizumab (Tysabri) and escitalopram (Cipralex).

## Methods

### Dataset

The data used in the analysis were collected by the Institute for Studies of the Medical Profession (LEFO) in the autumn of 2010. A questionnaire was sent by post to a representative panel of 1 543 Norwegian physicians. The respondents are part of a panel which every other year since 1994 has responded to a questionnaire concerning different aspects of how it is to be a medical doctor. The panel is drawn from the membership database of the Norwegian Medical Association (NMA), which in 2012 include a total of 24 718 physicians, constituting 95% of all physicians practising in Norway. In 1993, 2 000 randomly selected physicians were invited to be part of the reference panel, and 1 300 agreed. Since then two groups of younger doctors have been added to the existing panel to compensate for retirements and ensure the panel is representative [21].

The questions analysed in this study represent a subsection of the larger questionnaire. The subsection contained three parts; demographics, price estimates and questions about attitudes to costs. The questions were developed by reviewing existing literature and the cost pattern of Norwegian drug expenses. The questionnaire was tested on and discussed in detail with five physicians before it was sent out.

### Analytical variables

The dependent variable in this study is physicians’ knowledge of the prices of drugs. The questionnaire included five pharmaceuticals for which the respondents were asked to estimate the price. The physicians were asked to estimate the price of one year’s consumption of the standard dose of the pharmaceuticals. The price in question was the sum of the out-of-pocket price paid and the state reimbursement. Respondents were also asked not to check the prices when answering the questionnaire. We chose each specific drug on the grounds of total spending or the number of patients treated with it. We selected a combination of both expensive and inexpensive drugs. When asked for an estimate, respondents were given the medical condition to treat, the name of the drug and the standard dosage as recommended in the Norwegian catalogue of drug information, which is published by the Association of the Pharmaceutical Industry. The pharmaceuticals chosen were simvastatin (cholesterolaemia, 20 mg a day), alendronate (Fosamax) (osteoporosis, 10 mg a day), infliximab (Remicade) (rheumatoid arthritis, 240 mg every 8th week), escitalopram (Cipralex) (depression, 10 mg a day), and natalizumab (Tysabri) (multiple sclerosis, 300 mg infusion every 4th week). Estimates within 50% of the actual price were considered accurate, in line with similar studies [7,9,13]. Some studies accept as accurate only estimates within the range of 25%, but a wider range also eliminates variation or errors in obtaining the correct prices. Correct prices were obtained from the catalogue of drug information for pharmaceuticals [22]. A sensitivity analysis was performed to investigate whether the results would be different when the 25% range was used as the dependent variable (Appendix S1 and S2).

The independent variables in the study are demographic, such as gender, age, medical specialisation and occupational position, and the physician’s attitudes towards using the costs to society as a decision factor when treating patients. Senior consultant is used for all specialties to indicate that the physician has more extensive budget responsibilities.

Attitudes towards costs were measured thus: We asked the respondents to rate ten statements on a Likert scale from 1 (totally disagree) to 5 (totally agree). Also a discrete-choice question was part of this section of the questionnaire. In the discrete choice question, the respondents were asked how much extra they were willing to spend on behalf of society in the following scenario: “A patient with osteoporosis needs to take tablets once a week. A similar treatment is injections that must be given twice a year in hospital. Both treatments are covered by the state but the injections cost more per year than the tablets. How much extra do you think society should pay under various conditions? 1. Patient wants the injections option because they want to avoid the trouble of taking the pills every week. Pills cause mild nausea when taken (once a week). 2. In addition to the patient’s wishes and the nausea, assume that the injection is 10% more effective in increasing bone mass than the tablets.” Physicians willing to pay more than the median estimate of EUR405 (rate NOK to EUR0.135, http://www.xe.com) for scenario 2 are coded as “1” and physicians willing to pay less than this amount are coded as “0”.

Attitudinal variables were designed to determine whether the physicians themselves consider societal costs to be a legitimate decision factor. The sentence “I consider the cost to society when deciding whether or not to initiate an intervention” is a general statement. The sentence “I can reduce my referral and prescribing costs without compromising my patients’ health” will give us an indication of whether the physicians themselves are aware that they are actually practising with slack. Whether or not the physicians spend time checking prices will also say something about whether or not they are willing to support their attitudes with action. We are expecting to find that those who agree that they check prices also estimate prices more accurately than those who say that they do not check prices.

Descriptive statistics and logistic regression in SPSS, IBM Statistics 20 were used to analyse the data. The numbers reported from the logistic regression model are odds ratios (ORs). The dependent variables are dichotomised from the questionnaire items stating the price estimates on each of the pharmaceuticals. All physicians who were off by more than 50% were coded as “1” on this variable and physicians who were within 50%, here defined as giving a correct estimate, were coded “0”. One model has been developed for each of the five pharmaceuticals. The independent variables with an asterisk are those used in the basic model. To avoid co-linearity, closely related variables are substituted in subsequent models. This means that there has only been one specialist variable and one attitude measure in each model. A manual forward stepwise selection process was used to fit the models. Results are adjusted for the physicians’ geographic region of residence, but as we only report statistically significant results, this variable is not included in the here. In the regression analysis, the sample was reduced to include only those who work in clinical practice (n = 740). Excluded respondents were physicians in research, hospital or national administration and retirees. We made this exclusion because we wanted information only from clinicians who actually make decisions about resource allocation.

## Results

### Response rate and descriptive statistics

After two mailings a total of 1 009 physicians responded, giving a response rate of 65%.

Sample demographics are reported in Table 1. The sample differs from the population to some extent. As of September 2012 the Norwegian Medical Association has registered 22 705 physicians working in Norway below the age of 67, which is the most usual age of retirement in Norway. Forty-six percent of them are female, leaving our sample with a lower proportion of female physicians than in the real population.

**Table 1:** Demographic description of the sample.

	Subsample (n=740)	Entire sample (n=1 009)
**Age**	%	%
< 40	23	21
40-50	26	25
50-60	30	30
> 60	19	24
**Sex**	%	%
Female	39	37
Male	61	63
**Specialty/position**	%	%
General medicine	30	27
Senior consultant	53	42
Internal medicine	25	28
Surgery	10	11
Psychiatry	12	13

The group we left out of the subsample is older than the entire sample of responses, which can be explained by the exclusion of retirees.

Key information about the pharmaceuticals and estimates included in the analysis is displayed in Table 2. Average estimates are below the actual price for the two most expensive pharmaceuticals, and exceed the actual price of the three less expensive drugs. Escitalopram (Cipralex) is, on average, more correctly estimated. More than every second physician on the panel deviates by more than 50% from the actual price, and more than 60% deviate from the actual price by more than 25%.

**Table 2:** Summary statistics, price estimates.

	Information about actual price and use			Survey results			
	Actual price, EUR* standard dose, one year	Total cost in Norway 2009 (EUR)	Nr of users in Norway (Norwegian Prescription Database 2009)	Average price estimated EUR (standard deviation)	Median estimate EUR	Proportion of the panel with estimates more than 50% off	Proportion of the panel with estimates more than 25% off
Natalizumab (Tysabri)	27 427	1 019 131	58	10 972 (16 093)	5 407	83	90
Infliximab (Remicade)	11 774	481 513	83	8 493 (12 089)	5 407	63	82
Escitalopram (Cipralex)	343	22 539 295	98 453	562 (820)	406	53	66
Alendronate (Fosamax)	323	5 774 331	52 029	1 050 (1 809)	541	65	82
Simvastatin	137	31 795 280	356 615	589 (1 082)	270	66	77

* Rate NOK to EUR0.135, date 11.09.2012, www.xe.com (total sample, n=1009).

When physicians were asked how often they check the price of a treatment they would like to offer a patient, 82% responded “never or 1-5 times last year”. Thirteen per cent responded “once a month last year”, while 4% responded “once a week or more during the last year”.

### Logistic regression analysis

The results of the logistic regression analysis are reported in Tables 3 and 4. All variables except age are dichotomised and given the values 1 or 0. Age is used as an ordinal variable with the four values below 40, between 40 and 49, between 50 and 59 and above 59. Older respondents were more likely to estimate the price of simvastatin accurately. Since age is used as an ordinal variable, the effect refers to every change in category. A physician in the age group 50-59 has a 19% lower relative likelihood of deviating from the actual price than a physician in the age group 40-49.

**Table 3:** Attitudes to costs in medical treatment as explicators for estimates deviating from actual price (n=740).

	Physician’s estimate deviated from actual price by ≥50%				
	Simvastatin OR (95% CI)	Alendronate OR (95% CI)	Infliximab OR (95% CI)	Natalizumab OR (95% CI)	Escitalopram OR (95% CI)
I consider the cost to society when deciding whether or not to initiate an intervention*	1.0 (0.7-1.5)	1.1 (0.7-1.6)	0.8 (0.5-1.2)	0.6 (0.4-1.0)*	1.0 (0.7-1.4)
I can reduce my referral and prescribing costs without compromising my patients’ health	0.8 (0.5-1.3)	0.4 (0.2-0.6)**	1.4 (0.9-2.3)	1.6 (0.8-3.2)	0.9 (0.6-1.5)
Willing to pay more than 405 to give the patient his/her preferred treatment; injection vs. pill§	1.4 (0.9-2.2)	1.6 (1.0-2.4)*	0.7 (0.5-1.0)	1.2 (0.7-2.1)	1.2 (0.8-1.8)
Estimate of escitalopram (Cipralex) differed from actual price by ≥50%*	1.5 (1.0-2.2)*	2.2 (1.5-3.3)*	1.0 (0.7-1.4)	1.9 (1.2-3.0)**	__

Variable names with an asterisk are part of the basic model. Odds ratios with two asterisks indicate significance at the 1% level and those with one asterisk at 5%. Attitude measurement coding: 1 = agree, 0 = disagree. OR > 1 if physicians who agreed with the statement were more likely to deviate from accurate prices than others; OR <1 if they were less likely to deviate.

§ Explanation provided: injection avoids the trouble of taking the pills every week. Pills cause mild nausea when taken (once a week). In addition the injection is 10% more effective in increasing bone mass than the tablets.

**Table 4:** Demographic characteristics and medical specialty as explicators for estimates deviating from actual price (n=740).

	Physician’s estimate deviated from actual price by ≥50%				
	Simvastatin OR (95% CI)	Alendronate OR (95% CI)	Infliximab OR (95% CI)	Natalizumab OR (95% CI)	Escitalopram OR (95% CI)
Male Sex*	1.2 (0.8-1.8)	1.5 (1.0-2.4)*	0.8 (0.6-1.2)	0.9 (0.5-1.5)	1.2 (0.8-1.7)
Older Age*	0.8 (0.7-1.0)*	0.8 (0.7-1.0)*	1.1 (0.9-1.3)	0.9 (0.7-1.2)	1.0 (0.8-1.2)
GP*	0.4 (0.3-0.6)**	0.5 (0.3-0.8)**	0.9 (0.6-1.4)	1.3 (0.7-2.1)	0.8 (0.5-1.2)
Senior consultant	3.1 (2.1-4.7)**	2.0 (1.3-2.9)**	1.0 (0.7-1.5)	1.1 (0.7-1.8)	1.1 (0.7-1.6)
Internal medicine	1.2 (0.8-1.9)	1.1 (0.7-1.7)	0.8 (0.6-1.3)	0.4 (0.2-0.6)**	1.2 (0.8-1.8)
Surgery	1.8 (0.9-4.0)	1.3 (0.6-2.8)	1.7 (0.8-3.5)	3.2 (0.9-10.8)	2.4 (1.2-5.0)*
Psychiatry	2.2 (1.1-4.4)*	2.2 (1.1-4.7)*	1.3 (0.7-2.6)	2.6 (0.9-7.6)	1.1 (0.6-2.1)

Variable names with an asterisk are part of the basic model. Odds ratios with two asterisks indicate significance at the 1% level and those with one asterisk at 5%. Variables coding: Sex: 1 = man, 0 = woman, Age categories: 1 = below 40, 2 = between 40 and 49, 3 = between 50 and 59 and 4 = above 59. OR >1 if physician category more likely to deviate from accurate prices than others; OR < 1 if category less likely to deviate.

### Sensitivity analysis

A sensitivity analysis was performed to investigate whether the results would differ when the 25% error range was used as the dependent variable. The model was the same as reported in Tables 3 and 4, but all estimates were considered wrong if they differed more than 25% from the correct price. The results of this analysis are reported in Appendix S1 and S2. A sensitivity analysis to test the difference in results between all respondents to the questionnaire (n 1009) and the subset of clinically active physicians (n 740) was also conducted. The main differences between these analyses and the main models in Tables 3 and 4 are explained in the following.

GPs have a lower relative likelihood of deviating from the actual price by more than 50% on the drugs simvastatin and alendronate in the model with all respondents and on simvastatin in the model testing the 25% error rate.

Physicians specialising in internal medicine are more likely to estimate the price of natalizumab correctly, persisting in all three models.

The effect of age on alendronate persists when the error interval is reduced to 25%, meaning older physicians are more likely to estimate the price of alendronate correctly.

In all three models the physicians who agree with the statement “I can reduce my referral and prescribing costs without compromising my patients’ health” err less on the price of alendronate than the physicians who disagree with this statement. When testing the effect determining a 25% deviation from the actual price the respondents who agree are less likely to deviate from the price of infliximab, suggesting that the physicians who know the price of this drug do not agree that they can reduce their costs.

Another result persisting in all models is the higher odds of senior consultants deviating from the actual price of simvastatin.

When we ran the same model on the entire sample we saw some changes in results. The effect of sex on alendronate is no longer present, but is found for the estimates of infliximab. When reducing the error interval to 25% off the correct price, the effect of sex disappears entirely (see Appendix S2).

In addition to these sensitivity analyses the model has been run using the original attitudinal variables with five values each instead of the binary variables. Dichotomising these variables did not appreciably change the effects.

## Discussion

Physicians in general have poor knowledge of the cost of treatments they offer their patients. At first glance, it appears that physicians are almost guessing at random when they estimate prices for the treatments referred to in the questionnaire. More than 50% of the respondents inaccurately estimated the price.

Our findings as reported in Table 2 are similar to other studies suggesting that prices of cheaper drugs are overestimated while prices of more expensive drugs are underestimated [9,12,13,15,23]. This may lead to an overuse of resources because the cost difference is perceived by the doctor as smaller than it is, and will thus play a lesser role in decision-making than is justified when the doctor must choose between a more expensive product that is marginally more effective and a product that is cheaper [13]. Ryan et al. emphasise that overestimating the cost of cheap drugs and underestimating the cost of expensive ones may bias “practitioners’ choices towards higher cost products, thus inflating nations’ drug bills” [8]. Hoffman surveyed physicians’ knowledge of the cost of NSAIDs, and surprisingly found on average less knowledge of their most frequently prescribed drug than of other drugs; moreover, the most frequently prescribed drugs were underestimated more often. By declaring that it is not clear whether or not incorrect cost information influenced the physician to prescribe a particular medication more often, they introduce the possible explanation that physicians may prescribe some treatments more often because they erroneously believe them to be less costly [24].

The doctors who frequently prescribe a drug have better knowledge of its price than those who prescribe this medication less often. Ryan et al. conducted a study similar to ours in a sample of British physicians. They also asked physicians to indicate how frequently they prescribe each of the drugs for which they were asked for a price estimate. They did not find any correlation between knowledge of drug costs and the frequency with which the drugs were prescribed [8].

We also wanted to investigate whether a higher prescribing frequency was correlated with better price estimates. Since we lacked information about frequency of prescription of the different items, we have used the specialty of the physicians as a proxy. Natalizumab (Tysabri) to treat multiple sclerosis must be initiated by a specialist in internal medicine, which can explain why physicians specialising in internal medicine have 60% lower odds than other physicians of deviating from actual price by more than 50% (see Table 4).

We can argue along the same lines when attempting to explain the effect of being a GP on price knowledge. GP’s err considerably less often when estimating the price of simvastatin and alendronate (Fosamax). These two pharmaceuticals are typically administered by the primary care services, while infliximab (Remicade) and natalizumab (Tysabri) have to be initiated by a specialist, and are hence not a responsibility of the GP to the same degree. The effect of being a GP persists when we reduce the error range to 25%. One of the reasons why GPs remember the price of this drug in particular is that the cost of statins has been focused upon by governments for several years. In 2002 statins constituted 8% of total pharmaceutical sales in Norway [25]. The first generic versions of simvastatin were marketed in Norway from 2003, and since 2005 simvastatin has been the “preferred medicine” in the governments “first-choice product reimbursement scheme” for statins [5].

Male physicians have on average 50% higher odds of erring on the price of alendronate (Fosamax), compared with their female counterparts (see Table 4). Alendronate is used in the treatment of osteoporosis, a disease most commonly found in women. Female doctors more frequently have female patients [26], which might indicate that female physicians on average handle alendronate more often than do male physicians. A study also found that Norwegian female general practitioners are more restrictive in prescribing reimbursed drugs to patients above the age of 70 [27]. The difference in price knowledge between male and female physicians might in part be a result of the difference in the frequency with which the drug is prescribed by the two groups, e.g. as a result of gender differences in specialties.

The doctors who agree that cost is an important decision factor have better knowledge of the correct prices. Allan and colleagues reviewed 24 articles studying physicians’ knowledge of drug costs, finding that few factors impact the estimation of costs [12]. Our findings indicate that physicians who express cost-conscious attitudes deviate less when estimating prices. Those who claim they can reduce their own costs without harming patients deviate less frequently when estimating the prices on alendronate (Fosamax). Those who report to consider costs when treating their patients deviate less when estimating the price of natalizumab (Tysabri). Physicians who agree to pay a larger amount on behalf of society to achieve less nausea in patients receiving medication for osteoporosis deviate more than those doctors who are unwilling to accept such high spending for this effect. Interestingly the statistical effect is strongest for alendronate (Fosamax), which is an actual osteoporosis medication. It might imply that those doctors who actually treat these patients do not accept such a high price as those who do not treat osteoporosis patients.

### Implications

The results of this study indicate that physicians might not have the required knowledge to allocate resources efficiently. At the same time, the data also show that there is some potential to adapt and learn the prices of the pharmaceuticals most frequently prescribed by each doctor, especially when the physician perceives cost to be a relevant decision factor.

A fundamental question is whether physicians should have an obligation to consider efficient resource allocation in their role as therapists. Milton Weinstein asked in 2001: “Should physicians be gatekeepers of medical resources?” [28], and answers in the negative. Since their primary obligation is to be their patients’ advocate, Weinstein claims, “it is ethically untenable to expect doctors to face this trade-off during each patient encounter; the physician cannot be expected to compromise the wellbeing of the patient in the office in favor of anonymous patients elsewhere” (p 268).

On the other hand, the physician will have to make some form of resource allocation decisions in practice, whether she/he likes it or not. How much time to spend with a patient, should another test be ordered, or another consultation be booked – these are all choices that any doctor will make on the basis of professional discretion. Discretion in medical care is a necessary part of good medical work, thus the question is not whether the physicians should make allocative decisions, but on what grounds such decisions should be made. Clearly, a fair knowledge of the costs of treatment is one of the requirements in order to make ethically justifiable resource allocation decisions.

Lack of easily available information is one potentially important cause of the poor knowledge of costs. In a 1995 survey of Norwegian doctors, 51% said they did not have enough information about the cost of their daily decisions to be able to make good decisions about community resources [29]. If we acknowledge that physicians make choices which impact on how the total health-care resources are allocated between tasks and patients, and as such should be expected to choose cost effective treatments, they must be given the proper information and decision support tools. We see from other studies that physicians do not have good access to cost information, and often they believe that they could prescribe more cost effectively if they had access to better information [11,30,31]. In a recent study, Feldman, Shihab et al. concluded that displaying the prices of diagnostic tests can influence physicians ordering behaviour and lead to a reduction in costs [32].

### Methodological issues

Variations in the price of pharmaceuticals could constitute a problem because in this case there is no single true price. In Norway the regional variations are small because a maximum price is set centrally. There is some variation between the prices paid by hospitals, which have a separate pricing system, and the prices paid by others. However, the questions were worded to reduce this potential confusion by emphasising that price should be interpreted to include the payment by the patient, for the pharmaceuticals this is relevant for, and no patient pays for pharmaceuticals when they receive them in hospital.

The validity of the results depends on the representativeness of the sample. One potential problem might be that only those interested in economic issues answered the questions. Since the survey was part of a larger biennial survey, this problem is minimised, yet there is always the general problem that respondents may be more organised and knowledgeable than non-respondents. In this sense the results are conservative estimates of physicians’ knowledge of prices.

## Conclusion

Physicians have poor knowledge of the prices of pharmaceuticals. Some of our findings suggest that physicians who more frequently handle a treatment have better knowledge of the price of that treatment than other physicians. Expensive treatments are underestimated and less expensive treatments are overestimated. Finally, the data presented in this article suggests that physicians who agree that costs should be taken into consideration also have better knowledge of drug prices.

## Supporting Information

Appendix S1 **Sensitivity analysis, deviation from actual price greater than 25%.** Attitudes to costs in medical treatment as explicators for estimates deviating from actual price (n=740).
(DOCX)

Appendix S2 **Sensitivity analysis, deviation from actual price greater than 25%.** Demographic characteristics and medical specialty as explicators for price estimates deviating from actual price (n=740).
(DOC)
