# Supplementary material for: Norwegian Physicians’ Knowledge of the Prices of Pharmaceuticals: A Survey
Source: PLoS One. 2013 Sep 11;8(9):e75218. doi: 10.1371/journal.pone.0075218 (PMC3770612; doi:10.1371/journal.pone.0075218)
Supplement: Appendix S1 — Sensitivity analysis, deviation from actual price greater than 25%. Attitudes to costs in medical treatment as explicators for estimates deviating from actual price (n=740). (DOCX) [file pone.0075218.s001.docx]

Appendix S1. Sensitivity analysis, deviation from actual price greater than 25%. Attitudes to costs in medical treatment as explicators for estimates deviating from actual price (n=740).

|  | Physician’s estimate differed from actual price by ≥25% | | | | |
| --- | --- | --- | --- | --- | --- |
|  | Simvastatin OR (95% CI) | Alendronate OR (95% CI) | Infliximab OR (95% CI) | Natalizumab OR (95% CI) | Escitalopram OR (95% CI) |
| I consider the cost to society when deciding whether or not to initiate an intervention * | 0.9 (0.6-1.5) | 0.7 (0.4-1.2) | 0.7 (0.5-1.2) | 0.7 (0.4-1.2) | 1.1 (0.7-1.7) |
| I can reduce my referral and prescribing costs without compromising my patients’ health | 0.9 (0.5-1.5) | 0.5 (0.3-0.8) ** | 2.4 (1.2-5.1) * | 2.3 (0.9-6.2) | 1.0 (0.6-1.7) |
| Willing to pay more than 405 euros to give the patient his/her preferred treatment; injection vs. pill§ | 1.5 (0.9-2.5) | 1.4 (0.8-2.6) | 1.2 (0.7-2.0) | 1.1 (0.6-2.2) | 1.2 (0.8-2.0) |
| Estimate of Escitalopram (Cipralex) differed from actual price by ≥25% * | 2.1 (1.3-3.3) ** | 2.0 (1.2-3.4) ** | 1.2 (0.8-1.9) | 2.8 (1.5-5.2) ** | - |

Variable names with an asterisk are part of the basic model. Odds ratios with two asterisks indicate significance at the 1% level and those with one asterisk at 5%. Attitude measures’ coding: 1=agree, 0=disagree. OR > 1 if physicians who agreed with the statement were more likely to deviate from accurate prices than others; OR <1 if they were less likely to deviate.

§ Explanation provided: injection avoids the trouble of taking the pills every week. Pills cause mild nausea when taken (once a week). In addition the injection is 10% more effective in increasing bone mass than the tablets.
