# Supplementary material for: Norwegian Physicians’ Knowledge of the Prices of Pharmaceuticals: A Survey
Source: PLoS One. 2013 Sep 11;8(9):e75218. doi: 10.1371/journal.pone.0075218 (PMC3770612; doi:10.1371/journal.pone.0075218)
Supplement: Appendix S2 — Sensitivity analysis, deviation from actual price greater than 25%. Demographic characteristics and medical specialty as explicators for price estimates deviating from actual price (n=740). (DOC) [file pone.0075218.s002.doc]

Appendix S2. Sensitivity analysis, deviation from actual price greater than 25%. Demographic characteristics and medical specialty as explicators for price estimates deviating from actual price (n=740).

|  | Physician’s estimate deviated from actual price by ≥25% | | | | |
| --- | --- | --- | --- | --- | --- |
|  | Simvastatin OR (95% CI) | Alendronate OR (95% CI) | Infliximab OR (95% CI) | Natalizumab OR (95% CI) | Escitalopram OR (95% CI) |
| Male Sex* | 1.3 (0.8-2.1) | 1.4 (0.8-2.5) | 1.0 (0.6-1.6) | 0.8 (0.4-1.6) | 1.3 (0.8-2.0) |
| Older Age* | 0.8 (0.6-1.0) | 0.7 (0.5-0.9)** | 1.0 (0.8-1.3) | 0.9 (0.7-1.2) | 0.9 (0.8-1.2) |
| GP* | 0.5 (0.3-0.8)** | 0.7 (0.4-1.3) | 1.4 (0.9-2.5) | 1.9 (0.9-4.1) | 0.8 (0.5-1.2) |
| Senior consultant | 2.6 (1.6-4.2)** | 1.3 (0.8-2.2) | 0.8 (0.5-1.2) | 1.1 (0.6-2.0) | 1.2 (0.8-1.8) |
| Internal medicine | 0.9 (0.6-1.6) | 1.3 (0.7-2.5) | 0.6 (0.4-1.0) | 0.4 (0.2-0.7)** | 1.3 (0.8-2.0) |

Variable names with an asterisk are part of the basic model. Odds ratios with two asterisks indicate significance at the 1% level and those with one asterisk at 5%. Variables coding: Sex: 1=man, 0=woman, Age categories: 1=below 40, 2=between 40 and 49, 3=between 50 and 59 and 4=above 59. OR >1 if physician category more likely to deviate from accurate prices than others; OR < 1 if category less likely to deviate.
